# 超高效液相色谱-四极杆-飞行时间质谱法测定防脱发化妆品中19种非法添加化学成分

**DOI:** 10.3724/SP.J.1123.2021.08019

**Published:** 2022-04-08

**Authors:** Yalei DONG, Shuijiao NIU, Yasen QIAO, Chuanfeng HUANG, Haiyan WANG, Lei SUN

**Affiliations:** 1.中国食品药品检定研究院, 国家药品监督管理局化妆品研究与评价重点实验室, 北京 100050; 1. National Institutes for Food and Drug Control, Key Laboratory for Researching and Evaluation of Cosmetics of National Medical Products Administration, Beijing 100050, China; 2.山东省食品药品检验研究院, 山东 济南 250101; 2. Shandong Institute for Food and Drug Control, Jinan 250101, China

**Keywords:** 超高效液相色谱, 高分辨质谱, 非法添加, 防脱发化妆品, 筛查, ultra-performance liquid chromatography (UPLC), high resolution mass spectrometry (HRMS), illegally added, hair loss prevention cosmetics, screening

## Abstract

建立了超高效液相色谱-四极杆-飞行时间质谱(UPLC-Q-TOF-MS)法,结合高分辨质谱数据库,用于快速筛查及定量分析防脱发化妆品中19种非法添加化学成分。实验比较了提取溶剂的影响,优化了色谱条件和质谱条件。采用ACQUITY UPLC BEH C18色谱柱(100 mm×2.1 mm, 1.7 μm)进行色谱分离,以乙腈和2 mmol/L甲酸铵水溶液(含0.05%甲酸)为流动相进行梯度洗脱,运行时间为20 min。在电喷雾电离源、正离子扫描模式下,采用MS^E^模式采集19种化合物的质谱信息。根据标准品的色谱保留时间、母离子和碎片离子精确质量数及丰度比,在UNIFI软件中构建了非法添加化学成分的筛查数据库。结果表明,19种化合物线性关系良好,检出限为0.025~0.05 μg/g,定量限为0.075~0.15 μg/g。以溶液型防脱育发剂和膏霜型防脱洗发水两种常见防脱发化妆品为代表性基质进行加标回收试验,在不同的添加水平下,19种化合物的平均回收率为68.6%~118.8%,相对标准偏差(RSD)为0.3%~10.3%。通过对比保留时间以及母离子和碎片离子的精确质量数,该方法可用于77批样品的筛查。最终两批育发剂产品中均确证检出米诺地尔和非那雄胺两种非法添加化学成分,并对米诺地尔的裂解途径进行了推测;采用基质匹配外标法定量,结果表明其中米诺地尔含量高达60 mg/g,非那雄胺含量高达0.31 mg/g,说明防脱发类化妆品中存在多种化学组分同时添加的情况,且添加的药物含量非常高,具有很高的安全风险。该方法操作简便、灵敏度高、重现性好,能够同时用于防脱发化妆品中多种类型非法添加化学药物的筛查和定量分析,为防脱发化妆品的安全监管提供了强有力的技术手段。

## Abstract (en)

Cosmetic products for hair loss prevention are often mixed with prohibited substances such as hormones, antibiotics, and forbidden pharmacologically active substances. Although drugs increase the efficacy of cosmetic products, they cause skin irritation and allergic reactions, upon long-term exposure. Given the increasing number of hair loss prevention cosmetics on the market, the need to guarantee product safety calls for efficient and reliable methods to identify illegal ingredients in these products. Chromatography combined with high-resolution mass spectrometry offers the advantages of high resolution and high throughout, thus being a powerful technique for simultaneously detecting illegal ingredients in cosmetics. In this study, an ultra-performance liquid chromatography-quadrupole-time of flight mass spectrometry (UPLC-Q-TOF-MS) method for detecting 19 illegal chemical components was established. Combined with the scientific database, a screening platform for hair loss prevention cosmetics was constructed. The effect of extraction solvent was investigated. The chromatographic and mass spectrometry conditions were optimized. Under the optimal conditions, separation was achieved within 20 min on an ACQUITY UPLC BEH C18 column (100 mm×2.1 mm, 1.7 μm). Acetonitrile and 2 mmol/L ammonium formate solution containing 0.05% formic acid were used as mobile phases for gradient elution. The 19 compounds were detected by positive ion electrospray ionization (ESI) in the MS^E^ mode. The chromatographic retention time, precursor ions, product ions of the target analytes, and abundance ratio were collected to construct a screening database with UNIFI software. The 19 compounds were well separated, with good linearity. The limits of detection (LODs) and limits of quantification (LOQs) were 0.025-0.05 μg/g and 0.075-0.15 μg/g, respectively. Hair lotion and shampoo, which are commonly marketed as hair loss prevention cosmetics, were selected as the respective matrices for the recovery experiment. The average recoveries of the 19 compounds ranged from 68.6% to 118.6%, and the relative standard deviations (RSDs) were 0.3%-10.3%. Then, 77 batches of cosmetic samples were detected and screened under the same conditions. The TOF-MS information, including the retention time, ion addition mode, mass-to-charge ratio of the parent ions and fragment ions, as well as the abundance ratio, were compared between the cosmetic samples and the standard MS information with UNIFI software. Finally, two batches of samples that were illegally adulterated with minoxidil and finasteride were identified. The ESI fragmentation pathway of the product ions from minoxidil was also proposed. The matrix matching external standard method was used to determine the amounts of minoxidil and finasteride in the two batches of hair lotion, and they were as high as 60 mg/g and 0.31 mg/g, respectively. This result revealed that multiple chemical components were simultaneously added to hair loss prevention cosmetics. Furthermore, the amount of the illegally added drug was very high, indicating high safety risk for consumers using such cosmetics. The present method has the advantages of simple operation, high sensitivity, and good reproducibility. It can be used for rapid screening and simultaneous quantitative analysis of various illegal chemicals in hair loss prevention cosmetics.

由于现代生活工作节奏加快、精神压力过大等因素,我国的脱发人群迅速增加。防脱育发产品的消费需求呈指数级增长,防脱发化妆品的市场规模也不断扩大。临床治疗脱发的药物主要有雄激素抑制剂、生物学反应调节剂、皮质类固醇等^[1]^。受盲目追求利润的驱动,一些不法生产者将以上化学药物添加到防脱发类化妆品中,使得这一类化妆品面临滥用药物的安全威胁。米诺地尔和螺内酯是临床降压和利尿药物,具有刺激毛根、促进毛发生长的副作用,因此常被非法添加至防脱发产品,但长期使用会导致多毛症^[2]^以及心律失常等不良反应。氢化可的松、曲安奈德和醋酸曲安奈德等糖皮质激素具有显著的抗炎、免疫抑制功效,对斑秃等快速起效,但滥用糖皮质激素会导致产生接触性皮炎和激素依赖性;孕酮、睾酮等性激素在一定程度上会促进毛发生长,但会干扰人体正常激素平衡;酮康唑等抗真菌药物能够减轻油脂分泌和脱发;非那雄胺是一种5α-还原酶抑制剂,临床上能够抑制头皮毛囊变少,但会导致性欲减退等^[3]^。《化妆品安全技术规范》(2015版)^[4]^明确规定米诺地尔、螺内酯、性激素、糖皮质激素和抗感染类药物为化妆品中禁用成分,并规定了相关的5个检测方法。但现有的检测方法覆盖不全,相对分散,操作繁琐。因此,需要建立能够实现防脱发化妆品中多种类型非法添加化学成分的高通量筛查方法。

目前,防脱发化妆品中非法添加的检测方法主要有气相色谱-质谱法^[5]^、液相色谱法^[6]^以及液相色谱-串联质谱法(HPLC-MS/MS)^[7,8]^等。与传统检测方法相比,超高效液相色谱与高分辨质谱联用技术具有更好的选择性和更高的分辨率,在化妆品中禁限用物质的检测方面具有更显著的优势。高分辨质谱可用于化妆品中9种抗过敏违禁药物的定量分析^[9]^和66种抗生素^[10]^的高通量检测。超高效液相色谱-四极杆-飞行时间质谱(UPLC-Q-TOF-MS)能够对准分子离子和碎片离子的精确质量数进行分析,不但对前端分离的要求更低,而且结合数据库技术,能够达到更高的质量精度和更强的检测通量,兼之强大的谱库筛查功能,能够克服传统检测方法的不足,是化妆品中功效成分、限用物质、非法添加化学物质筛查确证的强有力工具。Xi等^[11]^通过建立SPE-UPLC-Q-TOF MS法测定育发化妆品中人参和甘草类的功效成分,证明高分辨质谱具有高灵敏度和高确证性的特点。Luo等^[12]^利用QuEChERS-同位素稀释-LC-TOF-MS高通量筛查化妆品中86种糖皮质激素,通过方法学数据证明了高分辨质谱在定性和定量方面的技术优势。目前,尚未有防脱发化妆品中多种类型非法添加化学成分同时测定的高分辨质谱法,也缺乏相关的筛查方法用于防脱发化妆品的快速筛查。

因此,本研究选择了在防脱发化妆品中法规规定的性激素、糖皮质激素、抗感染类药物、生物反应修饰因子、利尿剂以及尚未有法规规定的非那雄胺、依立雄胺、度他雄胺等19种非法添加化学药物为研究对象,通过UPLC-高分辨质谱获得化合物的保留时间、一级母离子和二级碎片离子精确质量数及丰度比,构建非法添加化学物质数据库。通过建立筛查方法,将该数据库用于市售防脱发化妆品的快速筛查,同时利用定量功能,实现定性确证和定量分析的同时进行。该筛查技术的建立,可为化妆品中非法添加化学成分的测定提供有效解决方案。

## 1 实验部分

### 1.1 仪器设备

超高效液相色谱-四极杆飞行时间质谱仪(美国Waters公司),配有ACQUITY UPLC I Class超高效液相色谱仪,Xevo G2-XS Q-TOF型飞行时间高分辨质谱仪,以及UNIFI科学信息管理系统;G10型离心机(北京白洋医疗器械有限公司); Vortex-5涡旋振荡器(江苏省海门市其林贝尔仪器制造有限公司); KQ-700DE型超声波清洗器(昆山市超声仪器有限公司); AL204型电子天平(美国Sartorius公司)。

### 1.2 材料与试剂

米诺地尔、螺内酯、联苯苄唑、克霉唑、益康唑、氟康唑、灰黄霉素、酮康唑、咪康唑、萘替芬、孕酮、睾酮、甲睾酮、氢化可的松、曲安奈德、醋酸曲安奈德、非那雄胺、度他雄胺、依立雄胺标准品来自中国食品药品检定研究院及Dr. E公司;甲醇、乙腈、甲酸、甲酸铵均为质谱纯(美国Fisher公司产品)。

精密称取标准品各10 mg,加入甲醇溶解,配制成质量浓度为1 g/L的标准溶液备用,4 ℃避光保存。取适量标准储备液,用甲醇稀释成质量浓度为10 mg/L的标准中间液。分别取标准中间液适量,用乙腈稀释成质量浓度分别为2.0、5.0、10.0、20.0、50.0和100.0 μg/L的系列标准溶液。

### 1.3 实验条件

1.3.1 色谱条件

色谱柱:ACQUITY UPLC BEH C18(100 mm×2.1 mm, 1.7 μm);柱温:40 ℃;进样体积:3 μL;流动相A: 2 mmol/L甲酸铵水溶液(含0.05%甲酸),流动相B:乙腈;流速:0.35 mL/min;梯度洗脱程序:0~0.5 min, 2%B; 0.5~2.0 min, 2%B~30%B; 2.0~6.0 min, 30%B~40%B; 6.0~13.0 min, 40%B~70%B; 13.0~16.0 min, 70%B~98%B; 16.0~18.0 min, 98%B; 18.0~18.5 min, 98%B~2%B; 18.5~20.0 min, 2%B。

1.3.2 质谱条件

离子化模式:ESI^+^;采集模式:MS^E^模式;离子源温度:120 ℃;毛细管电压:1.0 kV;采样锥电压:20.00 V;脱溶剂气温度:550 ℃;锥孔气流:50.0 L/h,脱溶剂气流:1000.0 L/h; MS^E^低碰撞能量:4 eV; MS^E^高碰撞能量:15~50 eV;实时校正液:亮氨酸脑啡肽溶液(200 μg/L), ESI^+^模式下*m/z* 556.2771。

### 1.4 化学药物筛查谱库建立

配制19种化学药物的标准溶液(50 μg/L),进行UPLC-Q-TOF-MS测定,在正离子模式下,采用MS采集模式扫描分析,通过一级全扫描确定待测物母离子的精确质量数和保留时间。再采用MS^E^模式,即低碰撞能量为4 eV,高碰撞能量为15~50 eV时,获得各化合物的母离子和所有二级碎片离子精确质量数及丰度比等质谱数据。将各化合物的质谱数据导入UNIFI软件中^[13]^,汇总各化合物的保留时间、一级母离子和碎片离子的精确质量数,结合各化合物的名称、分子式、结构式mol文件等基本信息,在UNIFI软件中构建19种化合物的筛查谱库。表1中给出了19种化合物的基本信息、母离子信息和丰度最高和次高的两个碎片离子的精确质量数。同时在UNIFI软件中将所建立的19种化合物的谱库导入,输入各级标准溶液的浓度,设置匹配参数,建立分析方法。

**表1:** 19种化合物的UPLC-Q-TOF-MS参数信息

No.	Compound	Chinese name	Chemical formula	Precursor ion	t_R_/ min	Observed (m/z)	Fragment 1 (m/z)	Fragment 2 (m/z)
1	bifonazole (B)	联苯苄唑	C_22_H_18_N_2_	[M+H]^+^	7.15	311.1542	165.0699	243.1168
2	clotrimazole (C)	克霉唑	C_22_H_17_ClN_2_	[M-C_3_H_3_N_2_]^+^	6.98	277.0777	165.0699	241.1012
3	dutasteride (D)	度他雄胺	C_27_H_30_F_6_N_2_O_2_	[M+H]^+^	11.59	529.2293	461.2033	95.0837
4	econazole (ECZ)	益康唑	C_18_H_15_Cl_3_N_2_O	[M+H]^+^	8.59	381.0322	125.0153	89.0386
5	epristeride (EPS)	依立雄胺	C_25_H_37_NO_3_	[M+H]^+^	10.66	400.2851	344.2227	72.0425
6	finasteride (FNS)	非那雄胺	C_23_H_36_N_2_O_2_	[M+H]^+^	7.22	373.2852	305.2603	317.2237
7	fluconazole (FCZ)	氟康唑	C_13_H_12_F_2_N_6_O	[M+H]^+^	2.8	307.1111	220.0681	238.0786
8	griseofulvin (G)	灰黄霉素	C_17_H_17_ClO_6_	[M+H]^+^	6.03	353.0781	285.0524	165.0546
9	hydrocortisone (H)	氢化可的松	C_21_H_30_O_5_	[M+H]^+^	3.77	363.2161	109.0648	119.0491
10	ketoconazole (K)	酮康唑	C_26_H_28_Cl_2_N_4_O_4_	[M+H]^+^	5.54	531.1566	489.1455	495.1794
11	methyltestosterone (MT)	甲睾酮	C_20_H_30_O_2_	[M+H]^+^	7.23	303.2313	285.2213	109.0648
12	miconazole (MCZ)	咪康唑	C_18_H_14_Cl_4_N_2_O	[M+H]^+^	9.71	416.9934	158.9763	122.9996
13	minoxidil (MXD)	米诺地尔	C_9_H_15_N_5_O	[M+H]^+^	2.63	210.1355	164.0933	193.1327
14	naftifine (N)	萘替芬	C_21_H_21_N	[M+H]^+^	5.68	288.1743	141.0699	117.0699
15	progesterone (P)	孕酮	C_21_H_30_O_2_	[M+H]^+^	9.95	315.2326	109.0648	97.0648
16	spironolactone (S)	螺内酯	C_24_H_32_O_4_S	[M-CH_3_OS]^+^	7.91	341.2112	107.0853	181.1108
17	testosterone (T)	睾酮	C_19_H_28_O_2_	[M+H]^+^	6.45	289.2164	97.0648	109.0648
18	triamcinolone acetonide (TA)	曲安奈德	C_24_H_31_FO_6_	[M+H]^+^	5.09	435.2174	415.2115	397.2010
19	triamcinolone acetonide	醋酸曲安奈德	C_25_H_31_FO_8_	[M+H]^+^	8.28	477.2284	457.2221	321.1485
	acetate (TAA)							

### 1.5 样品的筛查确证和定量分析

市售防脱发化妆品共77批,包含洗发水、洗发露、护发素等的膏霜类产品,以及精华液、育发液、育发剂等溶液型产品。分别称取两种类型的化妆品样品0.2 g于10 mL具塞离心管中,加入5 mL甲醇,充分涡旋分散,加入甲醇至近刻度,超声提取10 min,静置至室温,用甲醇定容至刻度,以10000 r/min离心5 min。移取上清液,经0.22 μm微孔滤膜过滤待测。注入UPLC-Q-TOF-MS,在MS^E^模式下,采集每一份样品的高分辨质谱数据。在UNIFI软件中导入77批样品的质谱数据,利用建立的数据库和分析方法对样品中的19种化合物进行定性筛查和确证。确证后的目标物采用母离子通过外标法定量。

## 2 结果与讨论

### 2.1 提取条件优化

以洗发水类化妆品基质中加标的19种化合物为对象,考察了甲醇和乙腈两种常用有机试剂作为溶剂的提取效果。结果显示,在两种基质中,如图1所示,采用甲醇作为提取溶剂,19种化合物的回收率普遍高于乙腈,且绝大部分化合物的回收率均能达到80%以上。因此,最终采用甲醇作为提取溶剂。

**图1:**
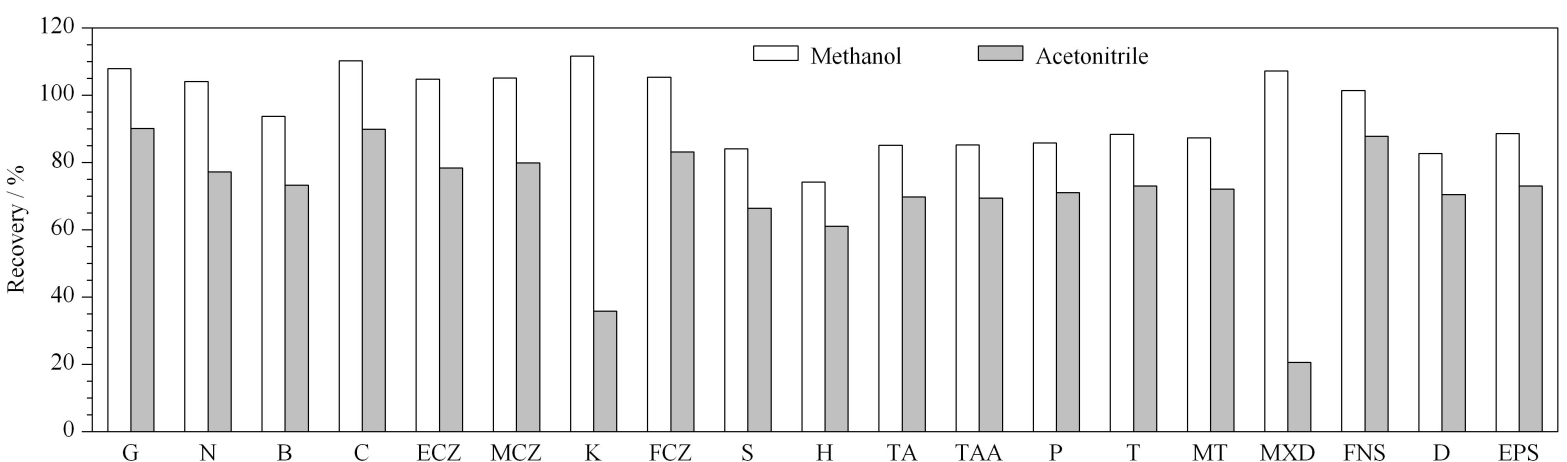
提取溶剂对19种化合物加标回收率的影响

随后,为进一步优化提取效率,探讨了提取溶剂中甲醇的含量对回收率的影响。配制甲醇体积分数为0、20%、40%、60%、80%、100%的甲醇水溶液,分别以其为提取溶剂考察对回收率的影响。结果(见图2)表明,对非那雄胺、氟康唑、氢化可的松、甲睾酮、米诺地尔、螺内酯、睾酮、曲安奈德和曲安奈德醋酸酯9种化合物,提取溶剂中甲醇的体积分数对回收率的影响不大。对于其他10种化合物,采用纯水作为提取溶剂,回收率很低,但是随着甲醇比例的提高,回收率显著增大。因此,为兼顾所有化合物的提取效率,采用纯甲醇作为提取试剂,使绝大部分化合物的回收率均能达到满意结果。该结论与文献及相关标准中采用的提取溶剂一致^[14]^。

**图2:**
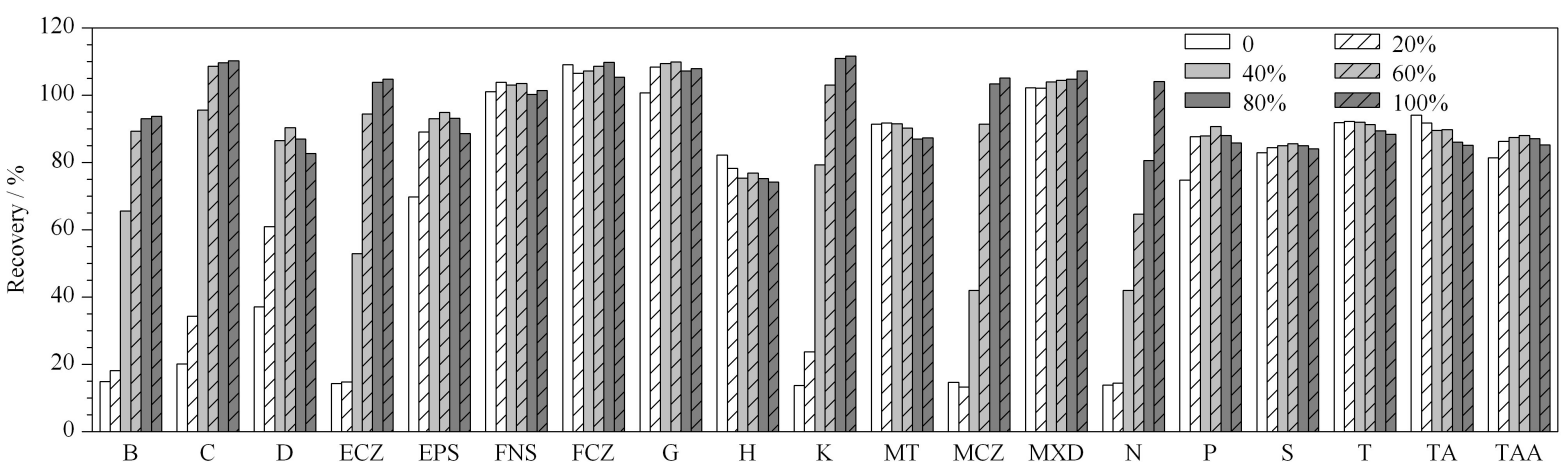
提取溶剂中甲醇的体积分数对19种化合物回收率的影响

### 2.2 色谱条件优化

根据19种化合物的结构特点,采用混合标准溶液,通过实验比较了ACQUITY UPLC HSS T3柱和ACQUITY UPLC BEH C18两种常用类型色谱柱的影响。结果(见图3)表明,采用HSS T3色谱柱时,保留较弱的米诺地尔和氟康唑两个化合物的峰形较差,而采用BEH C18柱时,19种化合物能够获得更好的峰形。进一步比较了50 mm和100 mm两种长度的色谱柱的分离效果。结果表明,采用100 mm的色谱柱时各化合物的分离度更好。

**图3:**
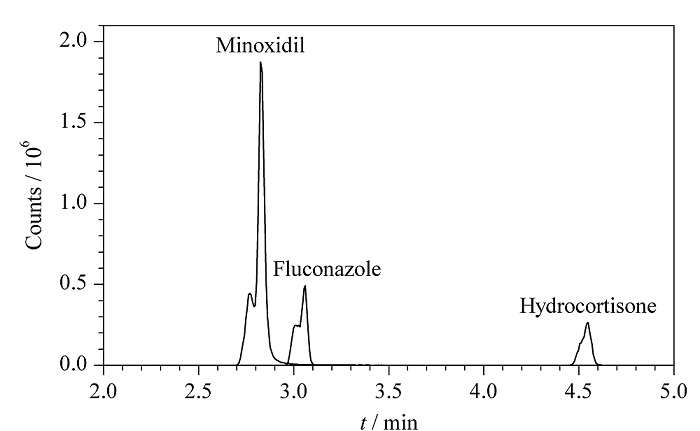
采用HSS T3色谱柱时部分化合物的提取离子色谱图

实验发现,由于该方法中的化合物种类较多,性质差异大,流动相添加剂对19种化合物分离的影响显著。结果表明,采用乙腈-水流动相体系,向水相中加入2 mmol/L甲酸铵和0.05%甲酸时,能显著改善米诺地尔和氟康唑两种化合物的峰形,且19种化合物均能获得较好的峰形和分离效果。这可能是由于甲酸铵可以改善含碱性基团化合物的峰形,而甲酸提供的H^+^不但能够提高化合物的离子化效率,与甲酸铵组成的缓冲体系使各化合物在流动相中的电离状态保持一致,有利于获得稳定的保留时间。因此,最终采用含0.05%甲酸的2 mmol/L甲酸铵-水溶液和乙腈分别作为流动相,以使19种化合物均获得较好的分离效果和灵敏度。实验结果表明,该液相色谱方法不但适用于正离子模式的化合物,对负离子模式的化合物同样适用。且方法具有可扩展性,能够根据需要不断添加所需要检测的药物,扩充所建立的数据库。进样溶剂与初始流动相中有机相比例相差较大会引起溶剂效应,造成峰形变差。通过实验尝试调整进样溶剂组成以减小溶剂效应,分别采用甲醇体积分数为0、20%、40%、60%、80%和100%的甲醇水溶液对混合标准溶液进行等比例稀释后进样。结果表明进样溶剂中甲醇和水的比例对19种化合物的保留时间和峰面积没有显著影响,且米诺地尔和氟康唑的色谱峰峰形得到改善;但当进样溶剂中含有水相时,氢化可的松、酮康唑、克霉唑以及益康唑、依立雄胺等几种化合物的峰顶均出现明显的分叉现象,如图4所示。因此,最终没有采用水相对进样溶液进行调整。采用更小体积的进样量是能够降低溶剂效应的手段之一。结果显示,当进样溶剂为甲醇、进样体积为3 μL时,各化合物均能获得相对较好的峰形,因此本方法中进样体积为3 μL。

**图4:**
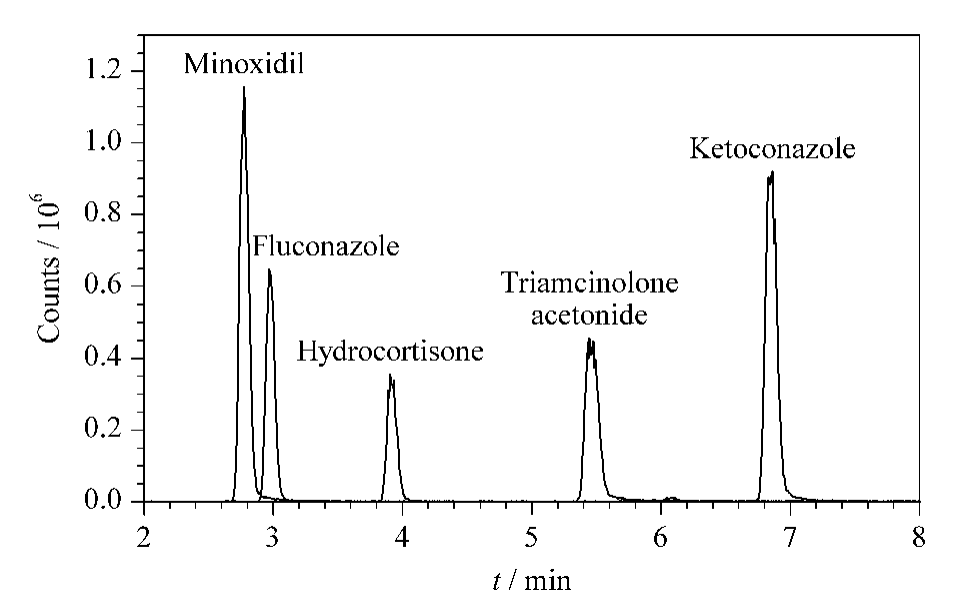
进样溶剂为60%甲醇-水时部分化合物的提取离子色谱图

### 2.3 质谱条件优化

首先,以各个目标化合物质量浓度为50 μg/L的单标溶液为测定对象,以UPLC-Q-TOF-MS的MS^E^采集模式进行扫描,通过优化锥孔电压来确定各化合物的母离子,推断一级母离子的加合形式。其中,克霉唑的母离子为其分子脱去一个咪唑基团形成的正离子峰,一级母离子的理论精确质量数为*m/z* 277.0777。螺内酯比较容易脱去乙酰基硫基^[15]^,生成*m/z*为341.2112的母离子。其他化合物的母离子均为[M+H]^+^峰的形式。随后在低碰撞能量为4 eV,高碰撞能量为15~50 eV条件下,进行二级碎片离子的质谱扫描,获得各组分的一级母离子和二级碎片离子精确质量数及丰度比。分别收集19种化合物的保留时间、母离子精确相对分子质量、各碎片离子精确相对分子质量、丰度比等信息,结合各化合物名称、分子式、结构式等信息,在UNIFI软件中建立19种目标化合物的质谱筛查谱库。图5a为最佳实验条件下19种化合物混合标准溶液的总离子流图,图5b为各化合物的提取离子色谱图。从图中可以看出,在最优实验条件下,19种化合物均能够获得较好的分离度和灵敏度,这有利于获得各化合物的准确质谱数据。

**图5:**
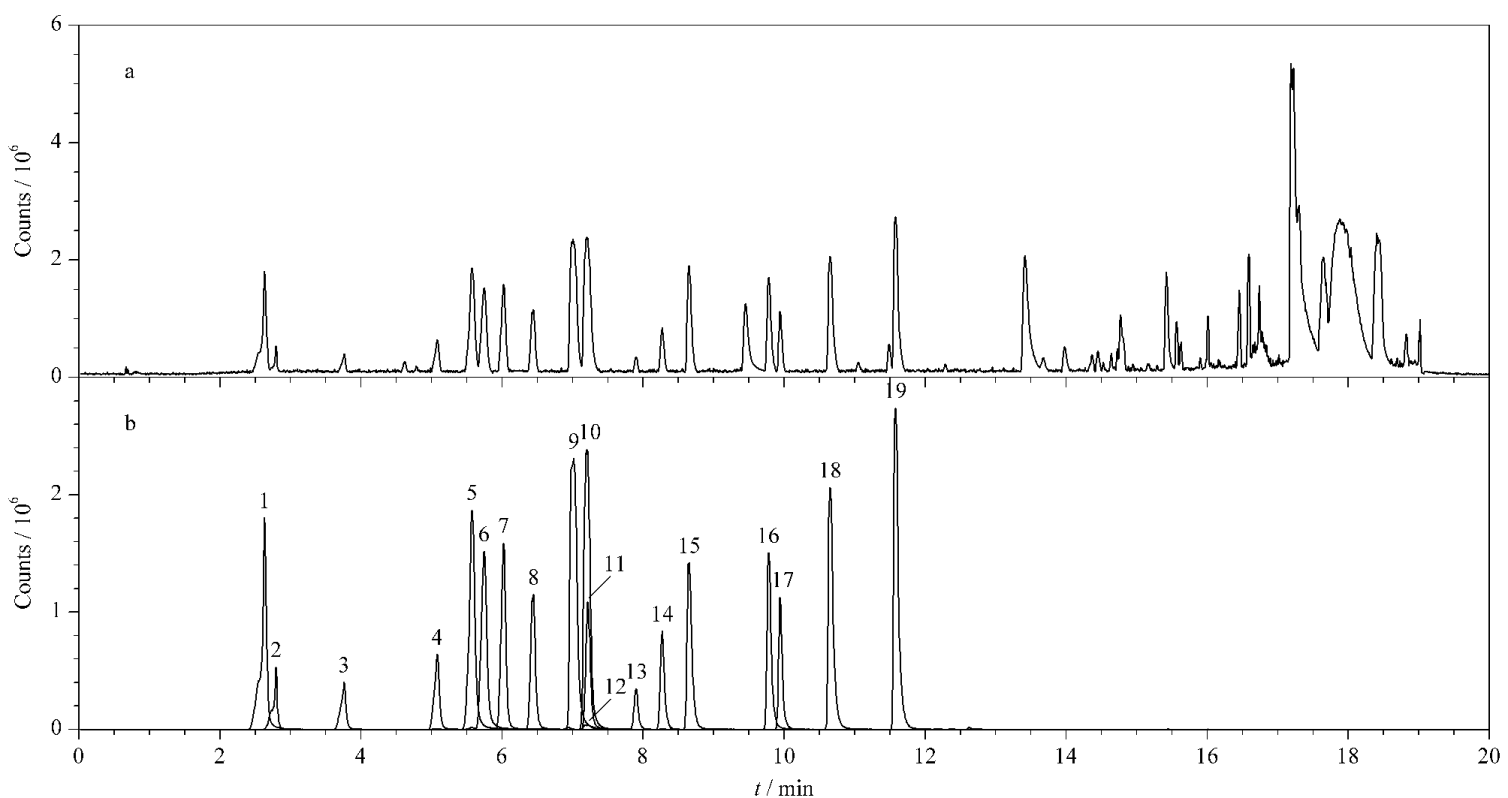
19种化合物的(a)总离子流图和(b)提取离子色谱图

### 2.4 方法学评价

2.4.1 基质效应考察

2由于化妆品基质复杂,与目标化合物共流出组分可能会影响电喷雾效率,对目标化合物的信号产生干扰,因此,基质效应在化妆品质谱分析中普遍存在。采用基质匹配标准曲线和溶剂标准曲线,以两者斜率之比考察了方法的基质效应。即ME=100%×基质匹配标准曲线的斜率/溶剂标准曲线的斜率。ME=100%,说明不存在基质效应,ME<100%,表示基质对分析物存在抑制作用。如图6所示,19种化合物在洗发水等膏霜类基质和育发液等溶液型基质中的ME位于50%附近且上下波动,说明两种基质中均存在不同程度的基质抑制效应。为克服基质效应,常用的方法有采用同位素内标、改进色谱分离、采用基质匹配标准曲线^[16]^进行校正等。本实验中分别选用膏霜状的洗发水和溶液状的育发剂作为代表性基质,采用基质加标法定量,以消除基质效应。

**图6:**
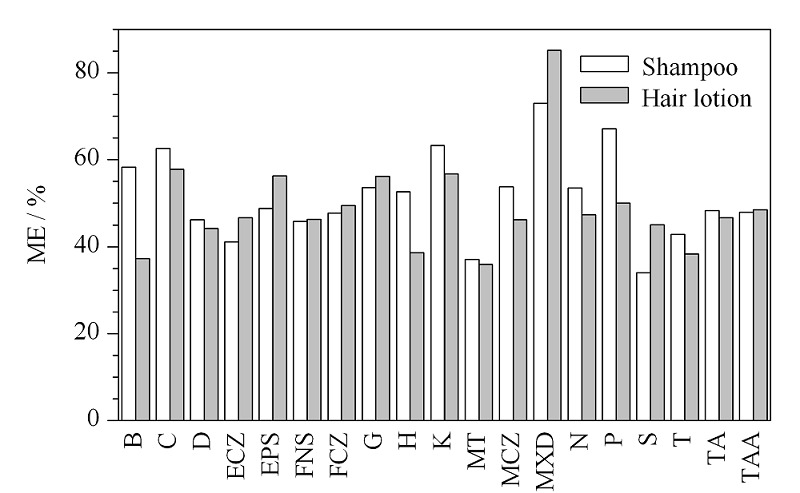
基质效应考察结果

2.4.2 线性关系、检出限、定量限与精密度

分别采用膏霜状的防脱发洗发水和溶液状的育发剂样品为空白基质,配制基质匹配标准溶液。结果表明,联苯苄唑的线性范围为5~50 μg/L,克霉唑、益康唑、度他雄胺、依立雄胺、非那雄胺、氢化可的松、甲睾酮7种化合物的线性范围为2~50 μg/L,其他11种化合物的线性范围为2~100 μg/L, 19种化合物在两种基质中的线性范围相一致,且相关系数(*R*^2^)均大于0.995(见表2)。依据《化妆品安全技术规范》(2015年版)理化检验方法总则^[4]^要求,将一定量混合标准溶液添加至空白基质样品中,根据样品检验操作方法进行处理,以信噪比分别为3、10时样品的质量浓度作为方法的检出限(LOD)和定量限(LOQ),其中联苯苄唑的检出限和定量限分别为1.0 μg/L和3.0 μg/L,氟康唑、米诺地尔和螺内酯的检出限和定量限分别为0.7 μg/L和2.0 μg/L,其他15种化合物的检出限和定量限分别为0.5 μg/L和1.5 μg/L。当方法取样量为0.2 g时,19种化合物的LOD和LOQ分别列于表2中。从表中数据可知,各化合物的检出限较低,能够用于膏霜和乳液类化妆品中19种化合物的灵敏测定。该方法中各化合物的检出限与《化妆品安全技术规范》(2015版)中相应的检出限相当甚至更低。因此,本方法具有更高的灵敏度。

**表2:** 两种基质中19种化合物的线性范围、相关系数、检出限、定量限和精密度

No.	Compound	Linear range/(μg/L)	R^2^	LOD/(μg/g)	LOQ/(μg/g)	RSD/%
1	bifonazole	5-50	0.9950	0.050	0.15	3.9
2	clotrimazole	2-50	0.9950	0.025	0.075	1.0
3	dutasteride	2-50	0.9995	0.025	0.075	3.2
4	econazole	2-50	0.9995	0.025	0.075	1.3
5	epristeride	2-50	1.000	0.025	0.075	3.6
6	finasteride	2-50	0.9991	0.025	0.075	1.1
7	fluconazole	2-100	0.9999	0.035	0.10	4.5
8	griseofulvin	2-100	0.9997	0.025	0.075	0.5
9	hydrocortisone	2-50	0.9999	0.025	0.075	3.5
10	ketoconazole	2-100	0.9991	0.025	0.075	2.3
11	methyltestosterone	2-50	0.9999	0.025	0.075	1.3
12	miconazole	2-100	1.000	0.025	0.075	2.2
13	minoxidil	2-100	0.9994	0.035	0.10	8.2
14	naftifine	2-100	0.9997	0.025	0.075	1.8
15	progesterone	2-100	0.9998	0.025	0.075	4.1
16	spironolactone	2-100	0.9991	0.035	0.10	5.9
17	testosterone	2-100	1.000	0.025	0.075	1.6
18	triamcinolone acetonide	2-100	0.9986	0.025	0.075	4.1
19	triamcinolone acetonide acetate	2-100	0.9974	0.025	0.075	4.6

采用空白样品进行基质加标(质量浓度为20 μg/L),在相同条件下平行处理6份进行测定,根据测定结果的相对标准偏差(RSD)计算方法的精密度(见表2)。表中结果显示,该方法的精密度小于8.2%,说明方法的精密度良好。

2.4.3 回收率

为验证方法准确度,在膏霜和溶液两种空白样品中进行19种化合物的加标回收率实验,每个浓度水平平行6份,计算平均回收率和RSD。从表3中可知,19种化合物在溶液型育发剂基质中的回收率为71.9%~118.8%, RSD为0.3%~9.3%,在膏霜类洗发水基质中的回收率为68.6%~117.4%, RSD为0.3%~10.3%。证明该方法灵敏、准确、有效,能够用于常见防脱发化妆品中19种非法添加化学组分的准确测定。

**表3:** 两种基质中19种化合物的加标回收率和RSD(n=6)

No.	Compound	Spiked/(μg/L)	Hair lotion matrix			Shampoo matrix	
			Recovery/%	RSD/%		Recovery/%	RSD/%
1	bifonazole	13.6	76.7	8.1		83.2	6.2
		27.2	86.2	3.6		77.4	2.1
2	clotrimazole	5.6	109.8	3.3		74.0	3.2
		11.2	110.7	0.9		87.4	5.7
3	dutasteride	10.1	71.9	4.8		82.5	4.2
		20.2	77.5	4.1		113.0	2.0
4	econazole	15.1	111.7	2.5		106.8	2.4
		30.3	107.6	0.7		76.9	9.8
5	epristeride	8.0	95.8	7.3		117.4	7.3
		16.0	117.5	3.4		81.5	7.3
6	finasteride	10.1	95.0	3.3		68.6	2.6
		20.2	93.8	0.3		99.8	3.2
7	fluconazole	6.8	101.2	5.5		71.9	2.2
		13.6	97.3	1.8		77.7	5.1
8	griseofulvin	6.2	88.6	2.5		94.4	0.9
		12.4	82.7	1.0		88.5	2.2
9	hydrocortisone	3.9	82.1	2.2		96.3	3.8
		7.7	118.1	5.2		114.1	8.5
10	ketoconazole	9.2	88.4	4.2		84.0	3.0
		18.4	83.8	0.6		94.4	4.6
11	methyltestosterone	9.8	116.9	6.1		84.4	3.4
		19.6	113.0	1.7		104.8	7.9
No.	Compound	Spiked/(μg/L)	Hair lotion matrix			Shampoo matrix	
			Recovery/%	RSD/%		Recovery/%	RSD/%
12	miconazole	7.9	118.8	3.6		105.5	6.2
		15.8	113.8	0.6		89.1	5.0
13	minoxidil	4.9	113.7	5.0		78.9	10.3
		9.8	105.8	0.7		93.2	3.7
14	naftifine	4.3	103.7	2.4		74.1	3.2
		8.6	97.6	0.7		108.9	4.1
15	progesterone	9.7	116.3	6.3		95.5	7.2
		19.5	104.8	1.7		89.2	1.1
16	spironolactone	10.5	74.4	4.9		73.9	6.1
		21.1	97.7	1.0		80.8	9.6
17	testosterone	10.5	112.6	6.2		85.1	4.1
		21.1	104.1	1.2		114.7	6.0
18	triamcinolone acetonide	10.8	88.8	9.0		83.1	5.7
		21.5	100.4	3.0		100.0	6.5
19	triamcinolone acetonide acetate	4.9	79.2	9.3		77.9	0.3
		9.8	109.1	2.7		115.9	2.7

### 2.5 实际样品的分析

在流通环节采购防脱发化妆品共77批,其中包括35批精华液、育发液、育发剂等溶液型化妆品,以及42批洗发水、洗发露、护发素等膏霜类化妆品。经UPLC-Q-TOF-HRMS在MS^E^采集模式下进行全扫描,用UNIFI软件对样品中的母离子、碎片离子的精确质量数及保留时间与谱库进行匹配。欧盟2002/657/EC准则要求禁用物质的确证必须达到4个识别点;在高分辨质谱中,每个母离子的识别点规定为2.0,子离子的识别点为2.5^[17]^。因此,利用四极杆-飞行时间高分辨质谱,只需一个母离子和一个子离子即可完成对目标物质的确证。设置保留时间偏差为±0.2 min,母离子精确质量数偏差为5×10^-6^(5 ppm)以内以及其他必要匹配参数后,如图7所示,其中一款育发剂产品的提取离子色谱图中分子离子峰的保留时间为2.63 min,低能量通道中存在*m/z* 210.1358,对应米诺地尔的[M+H]^+^峰,且与谱库中的信息一致,同时,该产品在保留时间为7.22 min处的低能量通道中存在*m/z* 373.2852,对应非那雄胺的[M+H]^+^峰。初步判断其为阳性样品。

**图7:**
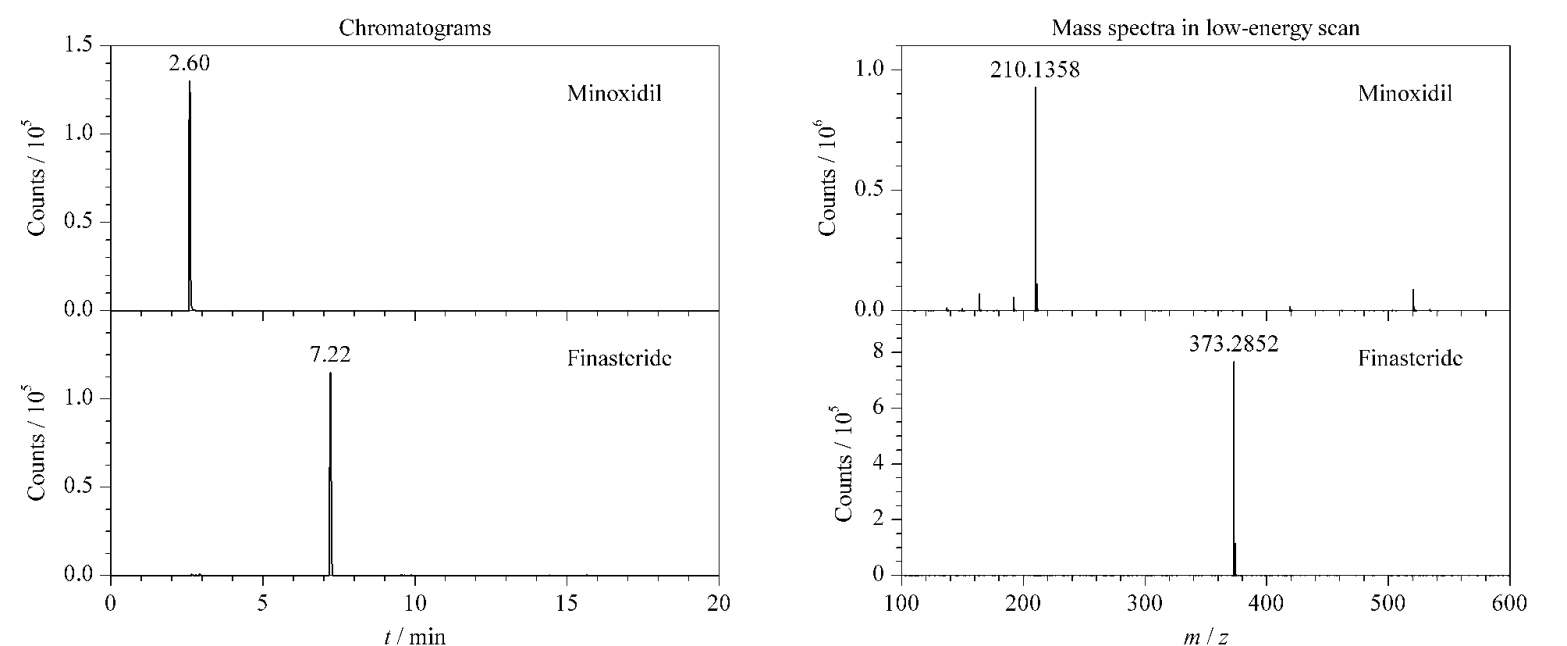
阳性样品中米诺地尔和非那雄胺的提取离子色谱图和低能量质谱图

将该款育发剂产品中检出的母离子和碎片离子与谱库中米诺地尔和非那雄胺标准品的结果进行比对,结果分别列于图8中。

**图8:**
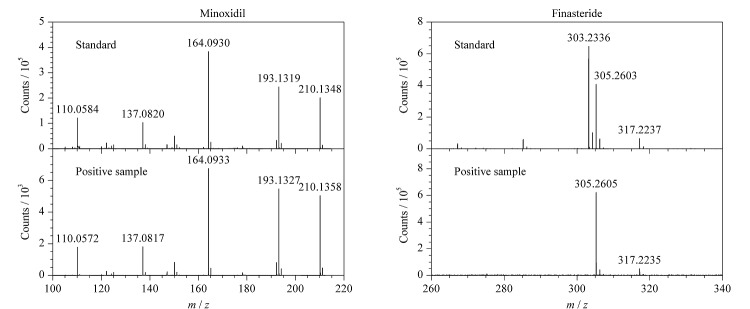
米诺地尔和非那雄胺的标准品及阳性样品的二级质谱图

通过与米诺地尔标准品的二级质谱图进行对比,高能量通道中既存在米诺地尔的分子离子峰,又存在碎片离子*m/z* 164.0933和*m/z* 193.1327,能够确证该产品中含有米诺地尔。通过与非那雄胺的二级质谱图对比,高能量通道检出碎片离子*m/z* 305.2605和*m/z* 317.2235,质量数偏差小于5×10^-6^。通过保留时间和母离子、碎片离子的精确质量数能够确证该样品中同时含有米诺地尔和非那雄胺。同样的方法能够确证另一款育发剂产品中也同时含有米诺地尔和非那雄胺两种非法添加化学成分。

以米诺地尔为例,推测其裂解途径如图9所示。米诺地尔的分子式为C_9_H_15_N_5_O,在正离子模式下获得质子,形成准分子离子,精确质量数为210.1358。经碰撞诱导,发生两种途径的裂解。一种途径是脱去一个氨基和哌啶环,形成C_4_H_4_N_3_O特征碎片离子(精确质量数为110.0354)。另一种途径是准分子离子脱去一个羟基,且哌啶环开环,形成C_9_H_15_N_5_(精确质量数为193.1327)的碎片离子,该碎片逐步脱去两个亚甲基,形成C_7_H_10_N_5_(精确质量数为164.0933)的碎片离子。该碎片离子进一步裂解,形成其他碎片离子,但相对丰度较小。因此,米诺地尔的二级碎片中相对丰度较高的碎片分别为*m/z* 164.0933(100%)、*m/z* 193.1327(84.7%)、*m/z* 110.0354(21.8%)。米诺地尔质谱裂解途径的解析,能够为其他类型化妆品样品中非法添加米诺地尔的快速分析和鉴定提供参考依据。

**图9:**
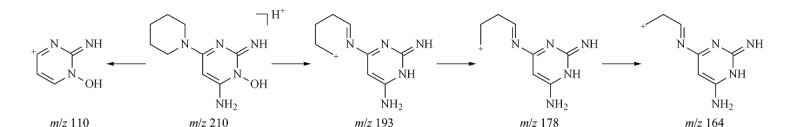
ESI模式下米诺地尔裂解途径推测

结合该方法的定量功能,对两批阳性样品中检出的米诺地尔和非那雄胺进行定量分析。两款产品中米诺地尔和非那雄胺的含量均很高,因此,对育发剂产品分别稀释250000倍和5000倍后进行测定。结果表明,两款育发剂产品中米诺地尔的含量分别高达60 mg/g和18 mg/g,非那雄胺的含量也分别为0.31 mg/g和0.19 mg/g。

以上定性和定量结果说明,防脱发化妆品中不但存在多种化学药物同时添加的情况,且添加的药物含量非常高。作为溶液型的育发剂,两款产品均注明需要在洗发后涂抹至头皮处至完全吸收,如此大剂量的化学药物在不知情的情况下被大量使用,可能会给使用者造成严重的副作用,严重危害消费者的身体健康^[18]^。

## 3 结论

建立了同时测定防脱发化妆品中19种非法添加化学组分的UPLC-Q-TOF-MS法,结合各化合物的质谱信息,构建了防脱发化妆品中非法添加化学组分的筛查数据库。该方法可用于防脱发化妆品中非法添加化学组分的筛查确证和定量分析,并最终在两批育发剂产品中检出了法规明确规定禁用的米诺地尔和法规外的化学药物非那雄胺。该方法前处理方式简单、灵敏度高、准确性好,分析流程高效,筛查结果准确,能够有效覆盖现有标准方法,大大提高了防脱发化妆品中非法添加的检测效率。可为化妆品安全监管提供高效解决方案,能够在各类非法添加突发事件和稽查应急检验中发挥优势作用。
